# Functional Characterization of Two Elongases of Very Long-Chain Fatty Acid from *Tenebrio molitor* L. (Coleoptera: Tenebrionidae)

**DOI:** 10.1038/s41598-017-11134-y

**Published:** 2017-09-08

**Authors:** Tianxiang Zheng, Hongshuang Li, Na Han, Shengyin Wang, Jennifer Hackney Price, Minzi Wang, Dayu Zhang

**Affiliations:** 1 School of Forestry and Biotechnology, Zhejiang A&F University, Linan, Zhejiang China; 2 The Key Laboratory for Quality Improvement of Agricultural Products of Zhejiang Province, College of Agricultural and Food Science, Zhejiang A&F University, Linan, Zhejiang China; 3 School of Mathematical & Natural Sciences, New College of Interdisciplinary Arts & Sciences, Arizona State University, Phoenix, AZ USA; 4 Zhejiang Dean Biotechnology Co. Hangzhou, Zhejiang, China

## Abstract

The elongases of very long chain fatty acid (ELOVL or ELO) are essential in the biosynthesis of fatty acids longer than C14. Here, two ELO full-length cDNAs (*TmELO1*, *TmELO2*) from the yellow mealworm (*Tenebrio molitor* L.) were isolated and the functions were characterized. The open reading frame (ORF) lengths of *TmELO1* and *TmELO2* were 1005 bp and 972 bp, respectively and the corresponding peptide sequences each contained several conserved motifs including the histidine-box motif HXXHH. Phylogenetic analysis demonstrated high similarity with the ELO of *Tribolium castaneum* and *Drosophila melanogaster*. Both *TmELO* genes were expressed at various levels in eggs, 1^st^ and 2^nd^ instar larvae, mature larvae, pupae, male and female adults. Injection of ds*TmELO1* but not *dsTmELO2* RNA into mature larvae significantly increased mortality although RNAi did not produce any obvious changes in the fatty acid composition in the survivors. Heterologous expression of *TmELO* genes in yeast revealed that TmELO1 and TmELO2 function to synthesize long chain and very long chain fatty acids.

## Introduction

Fatty acids (FAs) are molecules with a variety of biological functions including acting as energy sources and serving as components of cellular lipids and other molecules including eicosanoid hormones such as prostaglandins and leukotriences^1^. FAs are precursors of sphingolipids, glycerolipids^2^, hydrocarbons^3, 4^, fatty alcohols and wax esters^5^, which can participate in many cell biological processes such as reproduction, growth, migration, differentiation, and apoptosis and are components of pheromones in various arthropod species^6–8^.

Structurally, FAs are composed of long hydrocarbon chains that end in a carboxyl group and are classified based on the chain length and the number of double bonds. Fatty acids are roughly classified by length into the following groups: (1) Short Chain Fatty Acids (SCFA) which have five or fewer carbons, (2) Medium Chain Fatty Acids (MCFAs) which contain 6–12 carbons, (3) Long-Chain Fatty Acids (LCFAs), which contain more than 12 carbon atoms, and (4) Very Long-Chain Fatty Acids (VLCFAs), which contain 22 or more carbon atoms. Each class is associated with unique functions. For example, in mammals LCFAs act as ligands for peroxisome proliferator-activated receptors (PPARs) and regulate energy metabolism^9^ while VLCFAs have important anti-inflammatory roles^10^.

Fatty acid synthesis consists of a four-step cycle that includes condensation, reduction, dehydration and reduction steps and occurs primarily in the endoplasmic reticulum (ER)^11^. Each cycle extends an initial acetyl-CoA by two carbons and can be repeated up to seven times to form palmitic acid (C16:0)^2^. Further growth requires elongases of long or very long chain fatty acids (ELOVL or ELO) which can elongate C ≥ 14 fatty acids through a fatty acid condensation reaction. Elongases of very long chain fatty acids have been isolated from various organisms, including yeast, mammals, plants and other species^12–14^.

While ELOVLs have been relatively well characterized in vertebrates, little is known about these enzymes in insects, even though LCFAs and VLFAs are widespread among insect taxa^15–20^. Although some ELOVLs were isolated and characterized in a handful of arthropod species including *Drosophila melanogaster* and *Aedes albopictus* ^21^, the ELOs and ELOVLs in insects are poorly understood and require further investigation^4, 22, 23^. The yellow mealworm beetle, *Tenebrio molitor*, is a species that has abundant fatty acids including VLCFAs, yet the *T*. *molitor* elongases associated with VLCFA synthesis have not been identified. In this study, we identified and characterized two elongases (*TmELO1* and *TmELO2*) of Very Long-Chain Fatty Acids of *T*. *molitor*. These two ELOs were chosen out of the 20 putative ELOs identified from a transcriptome analysis due to ﻿the availability of full length sequences. Functions of *TmELO1* and *TmELO2* were analyzed using both an *in vitro* expression system in yeast and *in vivo* RNA interference in mature *T*. *molitor* larvae.

## Results

### Sequence Analysis of ELOs from *T*. *molitor*

The open reading frames (ORFs) of full-length *TmELO1* (GenBank accession no. MF279188) and *TmELO2* (GenBank accession no. MF279189) were identified by DNAStar and the physical and chemical properties of the deduced proteins were calculated using the ProtParam tool of ExPASy^24^ (Table 1). The instability index suggested that TmELO2 was a relatively stable peptide of 323 aa, and TmELO1 was of similar length at 334 aa but more unstable than TmELO2. The grand average of hydropathicity (GRAVY) shows that both TmELO1 and TmELO2 are hydrophilic. Further analysis indicates the presence of 5 putative transmembrane regions in TmELO1 (27–46, 66–88, 171–193, 205–224, 234–251 amino acids) and 7 for TmELO2 (25–47, 68–90, 116–135, 142–161, 171–193, 205–227, 237–254 amino acids). Prediction of subcellular locations using Euk-mPLoc 2.0^25–27^ suggests that both TmELO1 and TmELO2 were localized to the endoplasmic reticulum, the primary site of LCFA and VLCFA synthesis. Secondary structures of both TmELO1 andTmELO2 were predicted to be relatively similar to one another. Protein secondary structure prediction of TmELO1 showed the percentage of α-helixes, β-turns, random coils, and extended strands among the total amino acids to be 34.7%, 6.9%, 29.3% and 29.0%, respectively. The percentage in TmElO2 was 38.4% α-helixes, 8.1% β-turns, 23.2% random coils, and 30.3% extended strands.

**Table 1:** Sequence characters of TmELOs.

Name	ORF	Amino acids	MW	Theoretical pI	Instability index	GRAVY
TmELO1	1005	334	39200.6	9.31	41.55	−0.041
TmELO2	972	323	38357.8	9.29	35.06	−0.021

**Figure 1:**
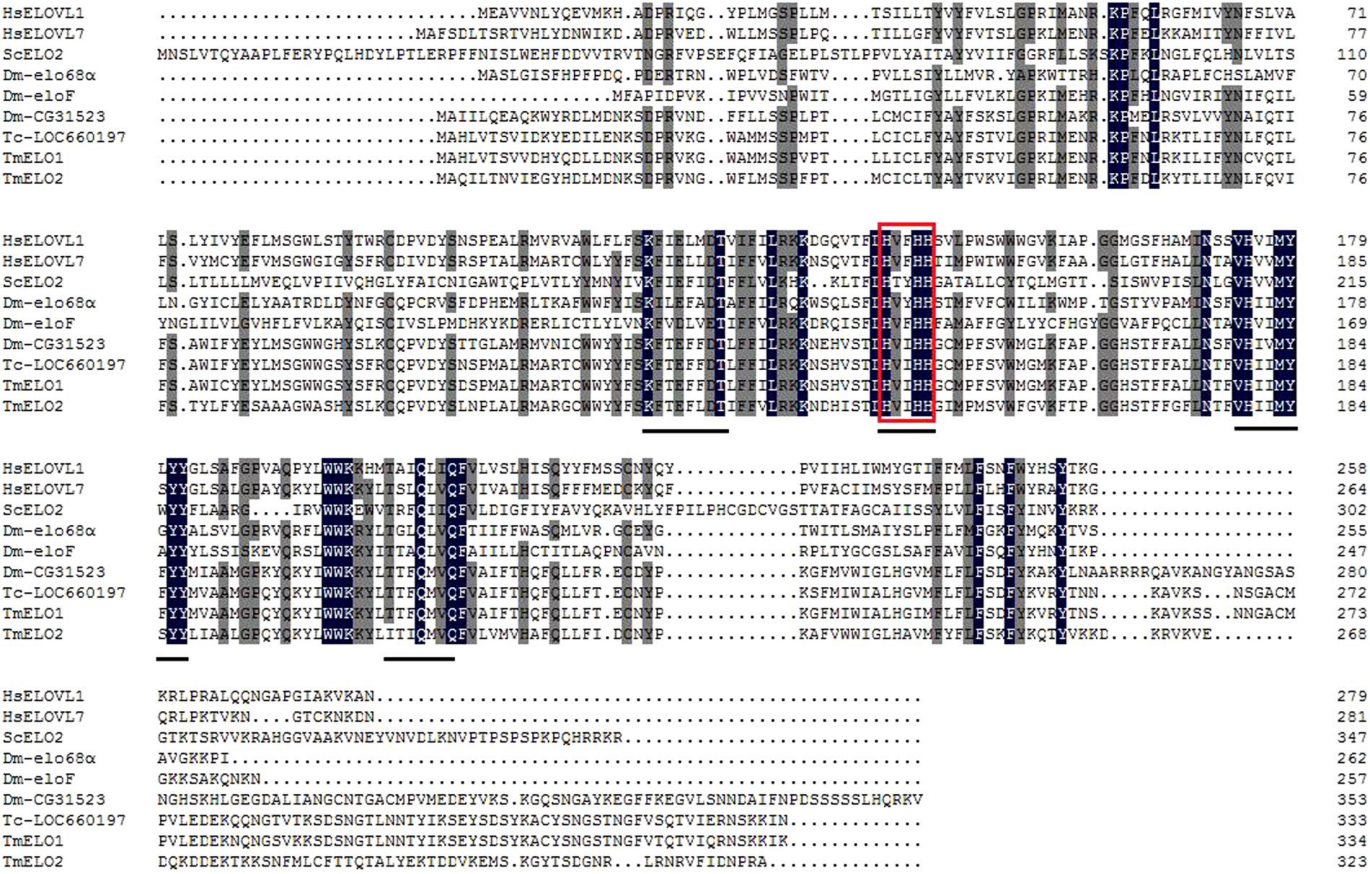
Multi-sequencing alignment of TmELO1 and TmELO2 with *H*. *sapiens* ELOVL1 and ELOVL7 (Genbank accession no. KJ894579.1 and AB181393.1), *S*. *cerevisiae* ELO2 (Genbank accession no. NM_001178748.1), *D*. *melanogaster* elo68α (CG32072), eloF (CG16905), CG31523 (Genbank accession no. AJ871925.1, AM292552.1 and NP_649474.1), *T*, *castaneum* LOC660197 (Genbank accession no. XM_966451.3). The similar motifs which were marked by black lines are distinctive signs to the ELONGASE protein families. The conserved histidine motif HXXHH in the centre of the protein is boxed.

Multi-sequencing alignment of the TmELOs with ELOs from other species showed that the proteins had some similar motifs such as KXXEXXDT, HXXMYXYY, TXXQXXQ and HXXHH, a histidine-box motif that is conserved in all elongases^28^ (Fig. 1). The TmELO1 and TmELO2 had the highest identity at 92.51% with *Tribolium castaneum* LOC660197 and 88.62% with *T. castaneum* LOC660257 respectively, two uncharacterized predicted elognases. Compared with* D. melanogaster* elongases, TmELO had identity at 60.17% with CG31523, an uncharacterized, predicted elongase in *D*. *melanogaster* (Genbank accession no. NP_649474.1). The TmELO sequences shared 17.18–43.79% identity with *Homo sapiens* with HsELOVL7 being the most similar. The TmELO sequences had low identity to *S*. *cerevisiae* with the highest identity being only 18.67%.

**Figure 2:**
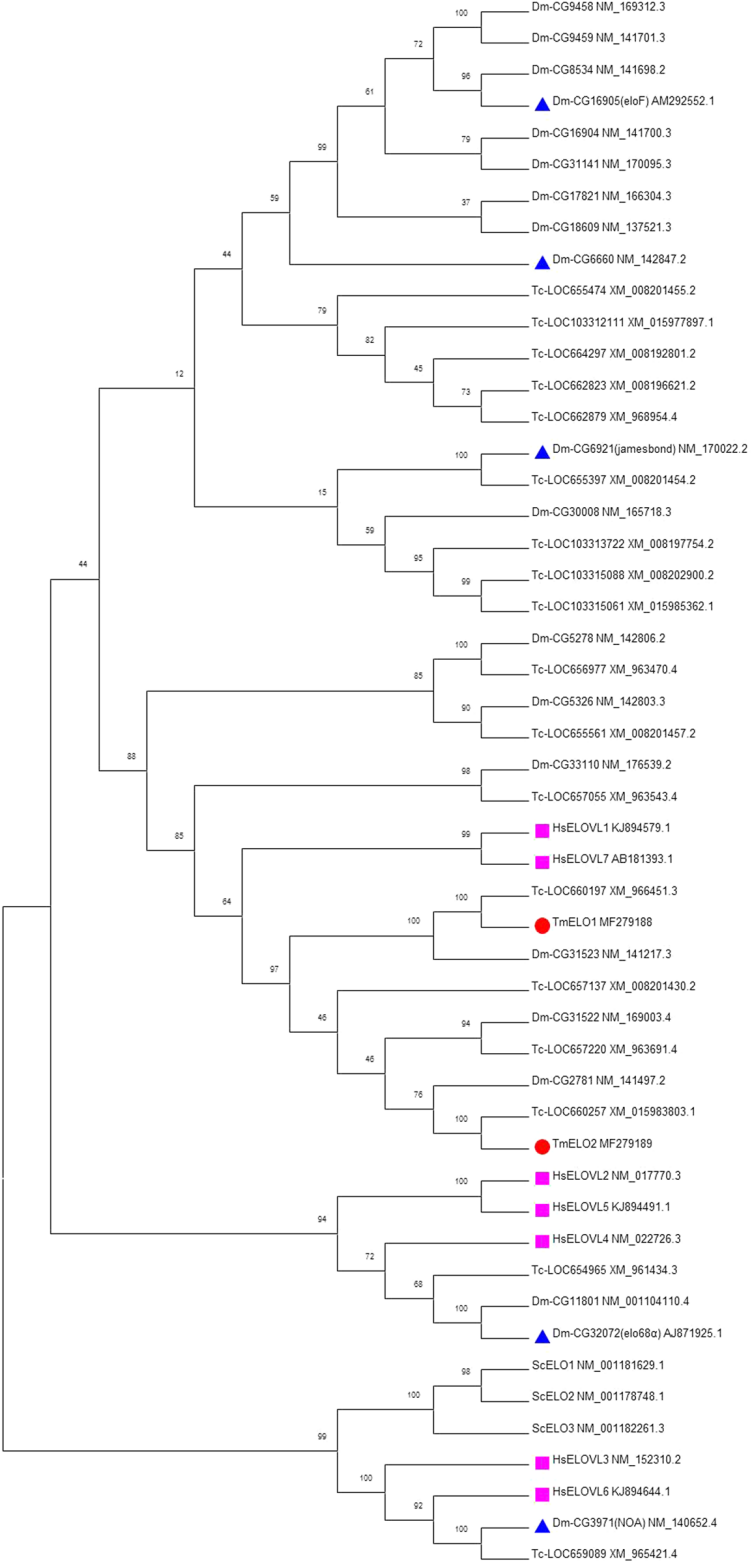
Phylogenetic tree of ELO included *H*. *sapiens* ELOVL1-7(marked by Purple squares), *S*. *cerevisiae* ELO1-3, all 20 *D*. *melanogaster* ELOs, 18 *T*. *castaneum* ELOs and TmELO1-2. Blue triangles indicate ELO which have been studied in insects. The phylogenetic tree was constructed using Neighbor-Joining method with 1000 bootstrap replicates. Each species was followed by its Genbank accession number.

A phylogenetic tree was constructed comparing the amino acid sequences of *T*. *molitor* elongases 1 and 2 with elongases from other organisms (Fig. 2). The phylogenetic analysis shows that TmELO1 clustered with *T*. *castaneum* LOC660197 and *D*. *melanogaster* CG31523 while TmELO2 clustered with *T*. *castaneum* LOC660257 and *D*. *melanogaster* CG2781, another predicted member of the ELO family.

### Relative transcript level at developmental stages

**Figure 3:**
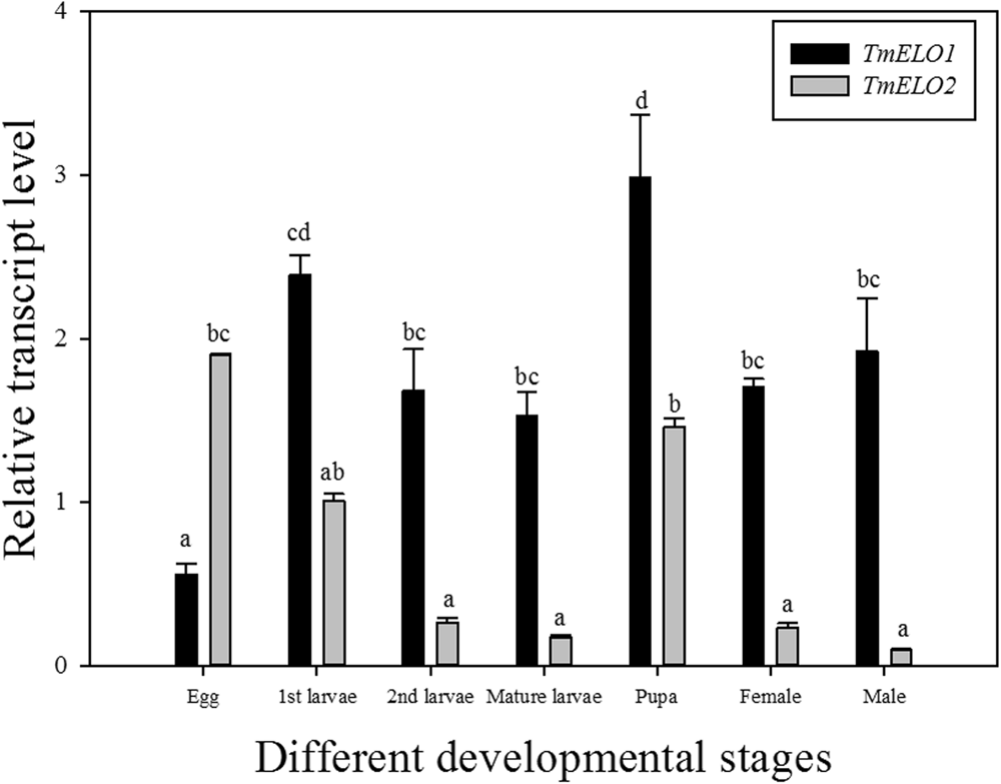
Relative transcript level of *TmELO1* and *TmELO2* at different development stages of *T*. *molitor*. The transcript levels of *TmELO1* and *TmELO2* at different developmental stages were conducted by qRT-PCR using RpS3 as a reference housekeeping gene. Y axis values are mean ± SE of relative expression levels; Lowercase letters (**a**–**d**) represent significant differences (*P* < 0.05) according to Tukey’s multiple range test.

Expression profiles of *TmELO1* and *TmELO2* at various developmental stages were generated using qRT-PCR (Fig. 3). Expression of both elongases was detected throughout all developmental stages examined. Expression of *TmELO1* peaked in 1^st^ instar larvae and pupae while *TmELO2* peaked in embryos and was somewhat reduced throughout the remaining developmental stages. During embryonic development, expression of *TmELO2* was significantly higher than *TmELO1*. Expression of *TmELO1* was significantly higher than *TmELO2* throughout all other stages examined.

### Fatty acid compositions and effects of RNAi

Total fatty acid compositions of mature larvae are listed in Table 2. The major fatty acids of *T*. *molitor* were C14:0, C16:0, C18:0, C18:1 and C18:2. Saturated fatty acids (SFAs), monounsaturated fatty acids (MUFAs) and polyunsaturaed fatty acids (PUFAs) were 25.55%, 32.26% and 41.25%, respectively. The fatty acids (C > 16) were 81.2%.

**Table 2:** Fatty acid compositions of mature *T*. *molitor* larvae.

Fatty acid	weight percent (n = 4)
C12:0	0.25 ± 0.02
C14:0	2.17 ± 0.04
C15:0	0.13 ± 0.00
C16:0	15.63 ± 0.19
C16:1	1.63 ± 0.05
C17:0	0.29 ± 0.01
C17:1	0.12 ± 0.01
C18:0	5.97 ± 0.16
C18:1	30.21 ± 0.36
C18:2	40.53 ± 0.34
C18:3	0.72 ± 0.01
C20:0	0.37 ± 0.01
C20:1	0.19 ± 0.00
C20:2	0.61 ± 0.11
C22:0	0.53 ± 0.01
C22:1	0.11 ± 0.02
C24:0	0.21 ± 0.06
Total SFA	25.55
Total MUFA	32.26
Total PUFA	41.25

Note: The values are shown the percentage of total FAs as means ± SE. n represents the number of independent samples.

**Figure 4:**
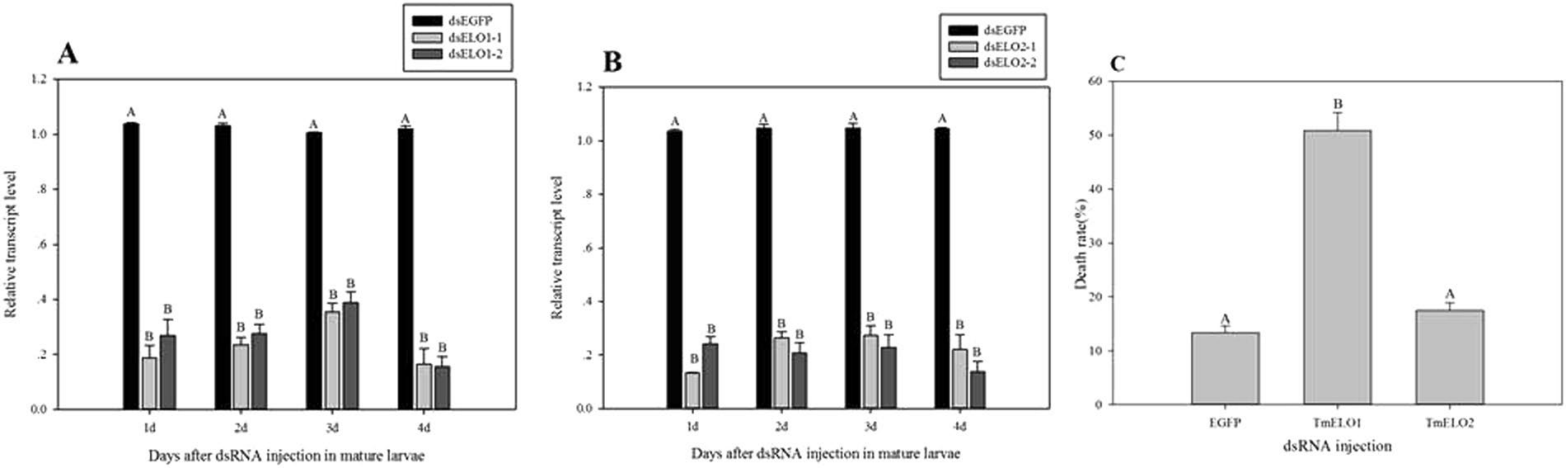
The dsRNA-mediated suppression of (**A**) *TmELO1* and (**B**) *TmELO2* transcrpts in mature larvae. 5000 ng dsRNA was injected in every larva. Students t test was used in data analysis. Y axis values are mean ± SE of relative transcript level. Uppercase letters (**A**–**C**) represent significant differences (P < 0.01) according to Tukey’s multiple range test. (**C**). Mature larvae of *T*. *molitor* were injected with dsRNA of EGFP or *TmELO1* or *TmELO2*. There were three replicates and each replicate included 40 mature larvae. Y axis values are mean ± SE of the death rate. Uppercase letters (**A**,**B**) represent significant differences (P < 0.01) according to Tukey’s multiple range test.

Double-stranded RNA (dsRNA) fragments were generated for gene silencing using RNA interference. The dsRNA fragment lengths of *TmELO1* were 361 bp (dsELO1-1) and 298 bp (dsELO1-2), and *TmELO2* were 401 bp (dsELO2-1) and 342 bp (dsELO2-2), respectively. The relative transcript levels of Tm*ELO1* and *TmELO2* in dsRNAs-injected mature larvae decreased significantly at 1d, 2d, 3d and 4d after injection (Fig. 4). One day after injection, the transcript level of *TmELO1* was significantly reduced by 76.5–81.3% and *TmELO2* was significantly reduced by 75.8–86.7%. Both *TmELO* genes were largely suppressed after 1d, 2d, 3d and 4d, which showed that the dsRNA had a long-lasting effects on the *TmELO1* and *TmELO2* expression. Fatty acid compositions in the survivors after RNAi injections were not significantly different than controls (data not shown), however, dsRNA injection was associated with increased larval mortality (Fig. 4). Compared to the control, mortality after *TmELO1* RNAi treatment significantly increased to 50.8%, while, the mortality following *TmELO2* RNAi treatment was slightly, but not significantly higher than controls.

### *In-vitro* functional characterization of TmELOs

**Figure 5:**
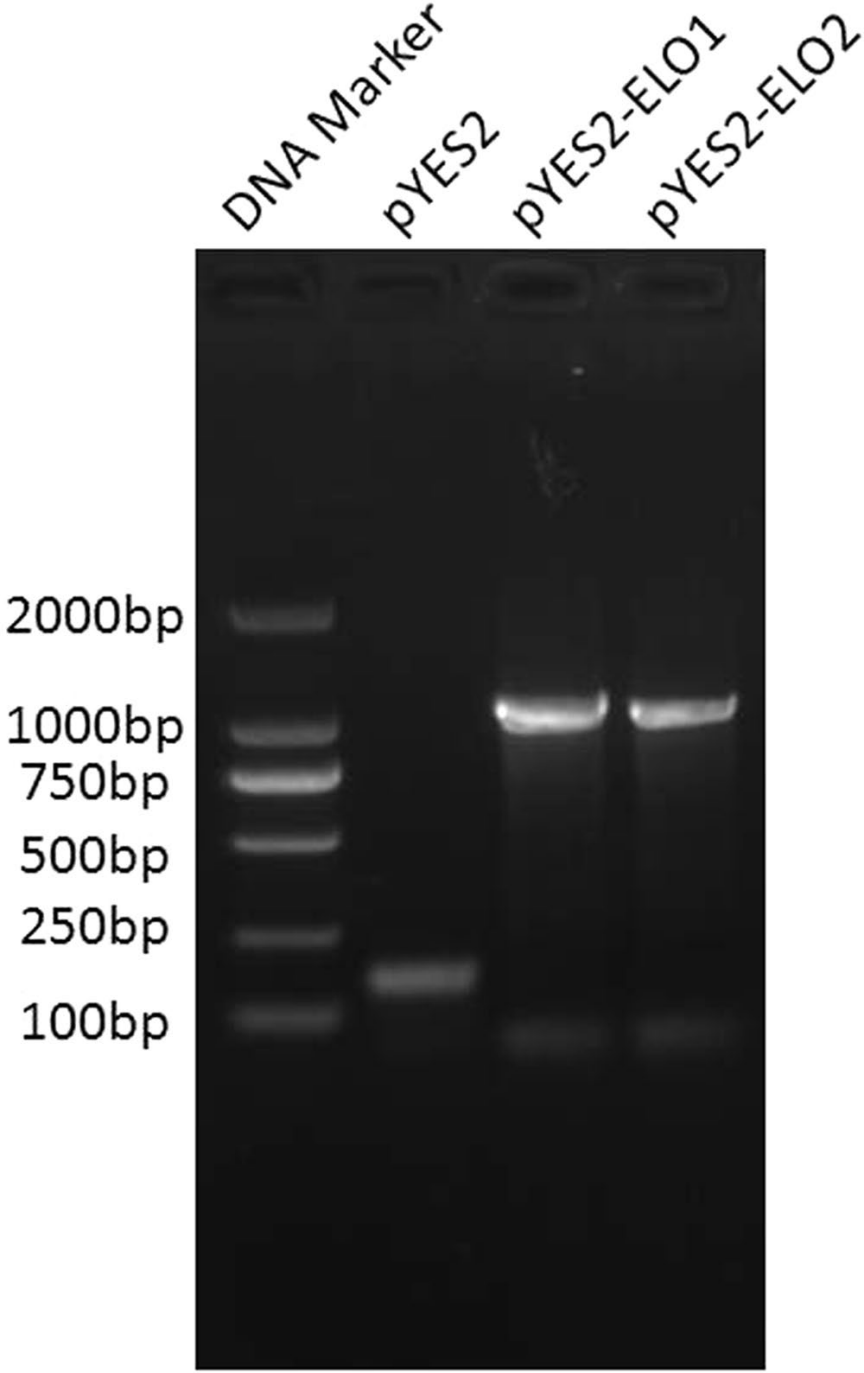
Reverse transcription (RT)-PCR analysis of *TmELO* transcripts in the transformed yeast lines. The RNA was isolated from the yeast that was transformed with recombinant plasmid and induced 24 h by galactose. PCR was conducted with CYC1 and T7 primers.

The ORFs of *TmELO1* and *TmELO2* cDNA were isolated from *T*. *molitor*, cloned into a pYES2 yeast expression vector, and the recombinant plasmids were introduced into yeast strain INVSc1. Transcripts of two TmELOs were detected by RT-PCR in the TmELO-transformed yeast (Fig. 5). The fatty acid analysis of transformed yeast showed *TmELO1* expression increased relative amounts of C14:0, C16:0, C16:1 and C 20:0, and C24:0 fatty acids and reduced C18:0 fatty acids when compared with controls (Table 3). Expression of *TmELO2* in yeast increased relative amounts of C14:0, C14:1, C16:0, C16:1 fatty acids and reduced C18:1 fatty acids. In general, our observations suggest that in yeast, TmELO1 could produce C20:0 and elongate SFA to C24; TmELO2 primarily increases the percentage of C16:0 and C16:1. (Table 3).

**Table 3:** Fatty acid contents in yeast INVSc1 with or without expressed *TmELO*s. Results are means ± SE (*n* = 4).

Fatty acid	Control(%)	TmELO1(%)	TmELO2(%)
C14:0	1.52 ± 0.04	2.12 ± 0.01*	2.03 ± 0.04*
C14:1	0.44 ± 0.00	0.79 ± 0.01*	0.55 ± 0.01*
C16:0	19.52 ± 0.15	20.04 ± 0.13	21.58 ± 0.33*
C16:1	41.65 ± 0.05	44.13 ± 0.19*	44.13 ± 0.23*
C18:0	8.28 ± 0.11	7.75 ± 0.17	9.46 ± 0.38
C18:1	28.38 ± 0.20	23.22 ± 0.16*	22.12 ± 0.43*
C20:0	n.d.	1.82 ± 0.03*	n.d.
C22:0	n.d.	Trace	n.d.
C24:0	n.d.	Trace	n.d.

*Values for contents of fatty acids in yeast transformed with TmELO1 or TmELO2 are significantly different from control as determined by the Student t test (P < 0.05). Trace, FA was detected at low levels, but not in all samples.

## Discussion

*Tenebrio molitor* larvae contain a large proportion of C ≥ 16 fatty acids including LCFAs and VLCFAs and here we characterize two elongases, *TmELO1* and *TmELO2* that are involved in their synthesis. Both *TmELO1* and *TmELO2* are expressed throughout the lifetime of *T*. *molitor* and do not exhibit sex-specific expression. During embryonic stages, *TmELO2* appears to be the predominant form while *TmELO1* is expressed at higher levels throughout the remaining developmental stages. Our results are consistent with findings in mammals that demonstrate developmental regulation of different elongases, and that expression is regulated not only by developmental signals but also by diet^29^. The effect of diet on *T*. *molitor* ELOVLs during development is intriguing but is outside the scope of the current study.

In the present study, expression of *T*. *molitor* elongases in yeast cells demonstrates differences in the fatty acids produced by TmELO1 and TmELO2 (Table 3). Expression of *TmELO2* led to significant increases of fatty acids up to 16 carbons in length, suggesting that TmELO2 function is limited to long-chain fatty acid synthesis. Meanwhile, *TmELO1* expression led to similar increases in long-chain fatty acids but also produced a significant increase in C20 fatty acids and slight, but not significant increases in C22, and C24 fatty acids, suggesting that TmELO1 may possess an additional role in the synthesis of very long-chain fatty acids. Surprisingly, RNAi-mediated knockdown of *TmELO1* and *TmELO2* in *T*. *molitor* did not significantly alter the fatty acid composition (data not shown). The lack of significant changes suggests that residual *TmELO1* and *TmELO2* transcript remained following RNAi treatment. It is also likely that *T*. *molitor* elongases have redundant roles and can maintain fatty acid levels in the absence of TmELO1 or TmELO2 activity. In support of the idea of redundant roles of elongases, ELOs in other species also have broad and somewhat overlapping functions. For example, in humans, HsELOVL1 can elongate C20–C26 SFAs^30^, while HsELOVL3 can elongate FAs from C16 to C22^2^.

Our observation that the two TmELOs generate FAs of different lengths is not unprecedented. In mammals, ELOVL1-7 play different roles in the elongation cycle of fatty acids^2, 31, 32^. ELOVL1 produces C20 to C26 SFAs and MUFAs^30^. ELOVL2 can elongate C20 to C22 PUFAs^33^. ELOVL3 can elongate FAs from C16 to C22, with the highest activity toward C18-CoAs^2^. ELOVL4 is specialized to elongate ultra long-chain fatty acids (ULCFAs) which are longer than C26^34, 35^. ELOVL5 elongates FAs from C18 to C20^36^. ELOVL6 can elongate C12:0 FAs to C16:0^37^. Finally, ELOVL7 has highest activity toward C18:3n-3 FA and C18:3n-6 FA^38, 39^.

Three ELOs of *S*. *cerevisiae* also showed variable functions. ScELO1 can elongate C14 FAs to C16 FAs^12^ while ScELO2 is involved in the synthesis of SFAs and MUFAs to C24 and ScELO3 is essential for the synthesis of C24:0 to C26:0 and can also elongate a wide range of SFAs and MUFAs^13^. Similar results were also observed in *Drosophila*. Chertemps *et al*. (2005) reported an elongase gene named elo68α in *Drosophila* males which can elongate myristoleic and palmitoleic acids (C14:1n-9 and C16:1n-9) *in-vtiro* expression in yeast^22^. Chertemps *et al*. (2007) found that eloF cDNA of *D*. *melanogaster* expressed in yeast could elongate medium-chain saturated and unsaturated fatty acids up to C30^4^.

In *T*. *molitor*, silencing of *TmELO1* via RNAi resulted in an increased mortality rate indicating that TmELO1 is essential for mealworm survival. Similar requirements for elongases in organism survival have been reported in other species. For example, in *Drosophila*, *RNAi to* CG6660, which encodes a predicted elongase, induced a similar lethal phenotype^40^. Elongases have also been shown to be required for neonatal survival in mice^41^, survival of the protozoan parasite, *Toxoplasma gondii* ^42^, and growth and survival of cancer cells^43^. Together, these studies emphasize that the requirement for elongases in growth, development, and survival is likely conserved among metazoans.

## Materials and Methods

### Insects

*T*. *molitor* were obtained from Shandong Agricultural University and have been reared in our laboratory at Zhejang A&F University for two years. The artificial climate chambers were maintained at 26 ± 1 °C, 65 ± 5% relative humidity and a 8:16 (L:D) photoperiod. The mealworm were reared on wheat bran mixed with cabbage.

### Cloning of *TmELO* cDNAs

Total RNA was extracted from different stages of *T*. *molitor* using RNAiso Plus (TAKARA, Dalian, China), per the manufacturer’s protocol and resulting RNA was stored at −80 °C. The concentration and quality of total RNA was measured with a UV/VIS spectrophotometer (BioDrop μLite, Cambridge, UK) and agarose gel electrophoresis was used to verify integrity of the RNA. The 500ng total RNA was used in cDNA synthesis reactions using the PrimeScript^TM^ 1st strand cDNA Synthesis kit (TAKARA, Dalian, China) and resulting cDNA was stored at −20 °C. *De novo* transcriptome sequencing was conducted at Tianke High-Tech Development Co., Ltd. (Zhengjiang, China) and the transcriptome analysis of *T*. *molitor* was performed in our laboratory (data not shown). The specific primers used to amplify two putative *ELO* genes are listed in Table 4. The PCR conditions for all these amplicons were 35 cycles of 94 °C for 15 s, 55 °C for 30 s and 72 °C for 1 min. A 3′A-overhang was generated by *Taq* DNA polymerase (TAKARA), and amplified products were gel purified with an E.Z.N.A gel extraction kit (Omega bio-tek, Norcross, GA) and linked with a plasmid pMD19-T vector (TAKARA) to form a recombinant plasmid, which was then transformed into *DH5α* (*E*. *coli*) for 10 hrs cultivation in LB solid medium with 50 μg/ml ampicillin. The plasmids were sequenced with sequence-specific primer M13 and maintained as glycerol stocks at −80 °C.

**Table 4:** Sequences of primers used in DNA cloning, dsRNA synthesis and qRT-PCR.

Primer name	Primer sequence (5′-3′)	Purpose
*TmELO1*-F	CTGTGCGGAAAACGGAAGAG	cDNA cloning
*TmELO1-*R	ATTTCGCCCCTGAGTTAC	
*TmELO2*-F	AGTGACTTGTGTTCTGTG	
*TmELO2-*R	AATTGCATTACGCCCGAGG	
e*TmELO1*-F	CGGGATCCATGGCTCACCTAGTTACCAG	yeast expression
e*TmELO1-*R	CCCTCGAGCTATTTGATCTTCTTTGAG	
e*TmELO2*-F	CGGGATCCATGGCACAAATATTAAC	
e*TmELO2*-R	CCCTCGAGTTACGCCCGAGGGTTGTCT	
ds*TmELO1*-F1	GATCACTAATACGACTCACTATAGGGGTGTCCAAACACTGTTCAG	dsRNA fragments
ds*TmELO1*-R1	GATCACTAATACGACTCACTATAGGGATTGCCGCCACCATGTAA	
ds*TmELO2*-F1	GATCACTAATACGACTCACTATAGGGCACGACCTCATGGATAAC	
ds*TmELO2*-R1	GATCACTAATACGACTCACTATAGGGTGGAGGGTCGAAATGTGG	
ds*TmELO1*-F2	GATCACTAATACGACTCACTATAGGGATGGCTCACCTAGTTACCAG	
ds*TmELO1*-R2	GATCACTAATACGACTCACTATAGGGGTTGACACCTGAAGCTGT	
ds*TmELO2*-F2	GATCACTAATACGACTCACTATAGGGCTAGATACGATCTTCTTCG	
ds*TmELO2*-R2	GATCACTAATACGACTCACTATAGGGTAGACCTATCCACCAGAC	
dsEGFP-F	GATCACTAATACGACTCACTATAGGGATGGTGAGCAAGGGCGAGGAGC	
dsEGFP-R	GATCACTAATACGACTCACTATAGGGGATCACTAATACGACTCACTATAGGG	
q*TmELO1*-F	TACTCATCAGTTCCAGCTTCT	qRT-PCR
q*TmELO1*-R	CACCGTTATTACTACTCTTCACA	
q*TmELO2*-F	CACGCCGTCATGTTCTACT	
q*TmELO2*-R	ACGCCCGAGGGTTGTCTAT	
q*TmRpS3*-F	GTGGTCGTTTCTGGCAAACT	
q*TmRpS3*-R	CAACACTCCTTGCCTCAACA	

Xho I site (GGATCC), BamH I site (CTCGAG) and T7 promoter sequences (GATCACTAATACGACTCACTATAGGG) are underlined.

### Sequence characterization of *TmELOs*

Full-length cDNA, *ELO* nucleotide sequences were translated into amino acid sequences and chemical and physical characteristics of Tm*ELO* genes were determined by the Expert Protein Analysis System program (http://web.expasy.org/protparam/)^24^. Trans-membrane structures were calculated using the TMHMM Server v.2.0 (http://www.cbs.dtu.dk/services/TMHMM/). Subcellular locations were predicted by Euk-mPLoc 2.0 (http://www.csbio.sjtu.edu.cn/bioinf/euk-multi-2/)^25–27^. SOMPA^44^ was used for the prediction of protein secondary structure. Amino acid sequences of other species ELOs were obtained from NCBI (http://www.ncbi.nlm.nih.gov/) and a phylogenetic tree was constructed and analyzed with respect to its homolog using the Neighbor Joining method. A bootstrap consensus tree of 1000 replicates was used to evaluate branch strength for analysis using MEGA 6.06.

### Preparation of dsRNA and injection

The dsRNA fragments of two *ELO* genes which were used to silence the corresponding mRNA transcripts were synthesized using the *in vitro* Transcription T7 Kit (TAKARA)^45^ and the specific primers used are listed in Table 4. Plasmid pMD19-T vectors linked with amplified *TmELO1* and *TmELO2* were the templates. The quality and length of the dsRNA fragments were measured by BioDrop μLite (BioDrop, Cambridge, England) and electrophoresis. The fragments were purified and diluted into a final concentration of 1 μg/μl. All dsRNAs were stored at −20 °C. Each of the two *ELO* dsRNA were injected separately into mature larvae of *T*. *molitor* and EGFP dsRNA was used as the control. The larvae were collected 1d, 2d, 3d and 4d after injection, flash-frozen in liquid nitrogen and stored at −80 °C. Animals were observed daily and the death rate was recorded through the pupal and adult stages till two weeks after injection. Every sample contained 20 larvae (n = 3) which were injected with 5 μg dsRNA fragments.

### Quantitative real-time PCR

Total RNA extraction from *T*. *molitor* and cDNA synthesis was conducted using the same procedures as Cloning of TmELO cDNAs. The cDNA products were diluted to 5 ng/μl. Primers (Table 4) for PCR and dsRNA synthesis were designed according to *TmELO1* and *TmELO2* sequences. The qRT-PCR reaction consisted of 20 μl including 10 μl SYBR^®^ *Premix Ex Taq* ^TM^ (TAKARA), 1 μl each of 10 μM forward and reverse primer, 1 μl of diluted cDNA and dd H_2_O. Ribosomal protein S3 (*TmRpS3*) (Genbank No.KJ868729.1) was selected as a housekeeping gene for normalization. Two-step qRT-PCR was conducted on Bio-Rad CFX96 (Bio-Rad, Hercules, California, USA) under the following conditions: 95 °C for 3 min, followed by 40 amplification cycles of 15 s at 95 °C and 30 s at 55 °C. Melting curves were used to verify the specificity of amplifications. Three biological replicates were carried out per treatment. Relative quantification analysis was then calculated using the 2^−ΔΔCt^ formula^46^.

### Heterologous expression of *TmELO* ORFs in yeast

The open reading frames (ORFs) of *TmELO1* and *TmELO2* were amplified by eTmELO-F and eTmELO-R primers. Primer F and Primer R separately contain a Xho I site and a BamH I site (Table 4). After PCR, the products were digested with Xho I and BamH I, ligated into the yeast expression vector pYES2 (Invitrogen), and used to transform *E*. *coli* DH5α. The *E*. *coli* were cultured and recombinant pYES2-TmELO1, and pYES2-TmELO2 plasmids were extracted from DH5α using a Endo-free Plasmid Mini Kit I (OMEGA) according to manufacturer instructions. The pYES2, pYES2-TmELO1, and pYES2-TmELO2 plasmids were introduced into INVSc1 (*Saccharomyces cerevisiae*) using the lithium acetate method^47^. A single colony was selected from INVSc1 strains transformed with pYES2 (as control), pYES2-TmELO1 or pYES2-TmELO2 plasmids, introduced into 15 ml of SC-U medium containing 2% glucose, and maintained overnight, with shaking, at 30 °C. The overnight cultured yeast was resuspended in 50 ml of SC-U medium containing 2% galactose and grown to an optical density (OD600) reached 0.4. Cells were harvested after 24 h at 30 °C with shaking, washed twice by dd H_2_O and stored at −80 °C until the FA were analyzed.

### FAs analysis

*T*. *molitor* larvae were homogenized and trans-methylated in 1% H_2_SO_4_ in methanol (v:v) at 80 °C for 2 h to prepare mealworm fatty acid methyl ester (FAME)(n = 4). Total lipid was extracted from yeast cells by using acidified glass beads to break cell wall for 10 min, and using chloroform:methanol (2:1, v-v) and 1% H_2_SO_4_ in methanol (v:v) at 80 °C for 2 h to prepare yeast FAME (n = 4). Mealworms and yeast FAME were added to 2 mL 0.9% NaCl and extracted twice with 2 ml of hexane. Hexane was subsequently removed by nitrogen gas and the total FAME was resuspended in 300 ml of hexane. After mealworms or yeast were homogenized, 100 μg C17:0 was added as an internal control. FAME were then extracted and analyzed by GC. The samples were analyzed on an Agilent 6890 N Gas Chromatograph (GC) equipped with a DB-23 column (60 m × 0.25 mm) with 0.25 μm film thickness. The following temperature program was employed: 160 °C for 1 min, then 10 °C/min to 240 °C, with He as carrier gas. F.A.M.E. Mix, C4-C24 (Supelco®) was used as the external standard.
